# The use of carvone in consecutive patch testing

**DOI:** 10.1111/cod.14249

**Published:** 2022-11-25

**Authors:** Johanna Enberg, Nils Hamnerius, Liv Kroona, Cecilia Svedman

**Affiliations:** ^1^ Department of Occupational and Environmental Dermatology Skåne University Hospital, Lund University Malmö Sweden; ^2^ Helsingborg's Hospital Helsingborg Sweden; ^3^ Department of Oral Pathology Malmö University Malmö Sweden

**Keywords:** contact allergy, fragrances, l‐carvone, oral lichen, patch test

## Abstract

**Background:**

Carvone (l‐carvone) is a mint‐tasting flavour additive that most of us is exposed to and can cause allergic contact reactions.

**Objectives:**

To analyse the frequency and the relevance of positive carvone reactions in a dermatitis population.

**Method:**

A retrospective analysis of dermatitis patients consecutively tested with carvone from 2017 to 2021. Data were retrieved from the department's patch‐test database.

**Results:**

Of 3554 patients tested with carvone, 28 (0.79%) had a positive reaction. Carvone‐positive patients had higher mean age, were significantly more likely female (*p* < 0.001) and had often an intraoral/lip involvement (*p* < 0.001). In the carvone‐positive group, 50% (*n* = 14) had a relevant reaction, and in 4 of 14, the relevance was first revealed after test reading. Of the carvone‐positive patients, 18 of 28 did not have a coexisting allergy to a fragrance/flavour allergen and of these 44% had a relevant allergy.

**Conclusions:**

The study suggests that a significant fraction of relevant carvone contact allergies may be overlooked if the allergen is not tested. Furthermore, as the exposure is widespread, inclusion of carvone in the Swedish baseline series may be justified even if the contact allergy prevalence is below 1%.

## Graphical abstract

Markers of fragrance allergy in the baseline series do not detect carvone allergy and an important fraction of relevant carvone contact allergies will be overlooked if carvone is not patch‐tested routinely.

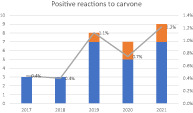

## 1 INTRODUCTION

Fragrance contact allergy is common in the general population and even more so in dermatitis patients. A group of fragrances is the cyclic terpenes to which the substance carvone belongs. Carvone (l‐carvone) is the primary component in spearmint and because of its mint‐aroma it is a flavour additive in several products, such as toothpaste, mouthwash, chewing gum, foods, beverages and flavoured tobacco products.^1^, ^2^, ^3^ Carvone is considered a weak allergen,^4^ but repeated daily exposure is very common, as most toothpastes contain l‐carvone.^5^ Spearmint oil production worldwide is estimated to be around 1500 tons/year and with a high annual consumption of nearly 90 tons in the United States.^6^, ^7^ Another terpene found in spearmint oil is limonene. Limonene is chemically related to carvone and carvone can be produced by oxidation of limonene.^8^, ^9^

Carvone contact allergy has predominantly been studied in small study populations, case reports and in the context of oral lichen. Studies on carvone in consecutively patch‐tested dermatitis populations are sparse. At our department, carvone has previously primarily been used in aimed testing. However, since 2017, carvone has been included in the department's extended baseline series. The main aim of this study is to investigate the occurrence of positive carvone reactions in a dermatitis population. A secondary aim is to characterize the carvone‐allergic group in terms of descriptive data and concomitant reactivity.

## 2 MATERIALS AND METHODS

### 2.1 Study population and data collection

All referred consecutive dermatitis patients above 18 years of age patch tested between January 2017 and December 2021 were included in the analysis. All data on patients were collected from the local database where age, sex, occupation, anatomical sites of dermatitis, patch test series, patch tests results and relevance were recorded.

### 2.2 Test series and allergens

All patients were patch tested with the Swedish baseline series and the departments' extended baseline series which includes the individual fragrances of the fragrance mixes, oxidized terpenes, carvone and gold. Carvone 5% were provided by Chemotechnique MB Diagnostics AB.

### 2.3 Patch testing

Patch testing was performed according to ESCD guidelines and test chambers were left at the patient's upper back for 48 h.^10^ Until the year 2017, the test chambers 8‐mm Finn Chambers (SmartPractice) were used, thereafter 8‐mm diameter Finn Chambers AQUA (SmartPractice) were used. A recent study showed no significant difference regarding detection of positive reactions between the two systems.^11^ Patch test results were read at Day (D) 3 or 4 and D7 according to ICDRG recommendations.^12^

### 2.4 Statistical analysis

For the analyses of concomitant allergy, three groups were examined: metals, preservatives and markers of fragrances found in the Swedish baseline series. Additionally, hydroperoxides of limonene and linalool were included in the fragrances group, due to their chemical kinship to carvone. Categorical data (sex, atopic dermatitis) and age were investigated with the Pearson chi‐square test or Fisher exact test when numbers were small. A *p* value of less than 0.05 was considered statistically significant. Uni‐ and multivariable logistic regression analysis was performed to estimate the crude odds ratio (OR) for anatomical site and concomitant contact allergies. The multivariable analysis was corrected for sex, age, atopic dermatitis and fragrance allergy. In case of missing data on history of atopic dermatitis, patients were excluded in the multivariate analyses. IBM SPSS Statistics for Windows (version 27.0; IBM Inc.) was used for statistical analysis.

### 2.5 Ethics

The study was approved by the Swedish Ethical Review Authority (Dnr 2020‐02190).

## 3 RESULTS

### 3.1 Prevalence

**FIGURE 1:**
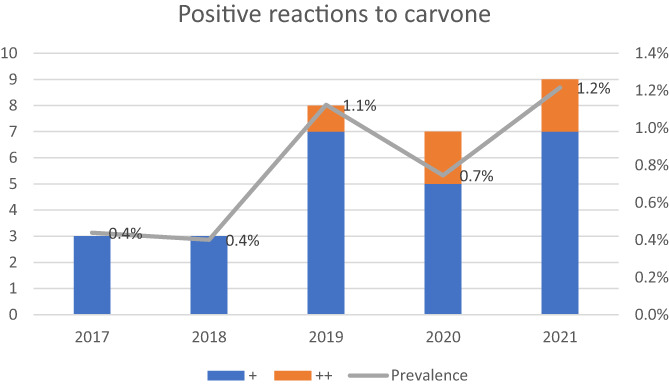
Trend of positive reactions to carvone. The colour of the bars corresponds to the strength of positive reactions to carvone.

Out of 3554 patients tested with carvone, 28 (0.79%) had a positive reaction. The prevalence was highest in 2021 (1.2%) and lowest in 2017 and 2018, 0.4%, respectively. The graph in Figure 1 indicates an increase in the prevalence of positive reactions of carvone during the years studied, although the trend did not reach statistical significance (*p* for trend 0.06).

### 3.2 Demographics

The carvone‐positive patients had a higher mean age, 60.2 years compared with 44.3 in the carvone‐negative group (*p* < 0.001) and were more likely female (96% vs. 68%, *p* < 0.001). No significant difference was found regarding history of atopic dermatitis (carvone‐positive 25% vs. carvone‐negative 29%).

### 3.3 Carvone patch test reactions

Twenty‐three of 28 patients (>80%) had a weak positive reaction (+), and 5 had a strong reaction (++). Five patients, that is, 17.8%, were positive on D7 only. There were 47 patients with a doubtful reaction and 2 patients with irritant reactions.

### 3.4 Clinical relevance

In the carvone‐positive group, the allergy was considered clinically relevant in 14 (50%) patients. A suspicion of fragrance and/or flavour allergy before testing was observed in 10 of these 14 patients. However, in 4 of these 14 patients (28%), a contact allergy to carvone was not suspected before it was detected at test reading.

### 3.5 Anatomical sites of dermatitis

Both intraoral/lip and anogenital/groin sites had a clear positive association to carvone allergy, while upper extremity site, including hands, was underrepresented in the carvone‐positive group. Additional affected anatomical sites are shown in Table 1. It was possible to record more than one site per patient.

**TABLE 1:** Association between contact allergy and anatomic site(s)

Contact allergy to carvone				
Site(s)^a^	Yes (*n* 27), *n* (%)	No (*n* 3009), *n* (%)	OR (95% CI)	
			Univariate	Multivariate^b^
Intraoral or lip	7 (26)	*114* (4)	8.9 (3.7–21.4)	5.5 (2.2–13.8)
Face	5 (19)	904 (30)	0.5 (0.2–1.4)	0.5 (0.2–1.2)
Head or neck^c^	3 (11)	451 (15)	0.7 (0.2–2.4)	0.6 (0.2–2.0)
Upper extremity	7 (26)	1653 (55)	0.3 (0.1–0.7)	0.4 (0.2–1.1)
Lower extremity	4 (15)	324 (11)	1.4 (0.5–4.2)	1.5 (0.5–4.4)
Trunk	2 (7)	345 (12)	0.6 (0.1–2.6)	0.5 (0.1–2.3)
Anogenital or groin	5 (19)	56 (2)	12.0 (4.4–32.8)	9.6 (3.2–28.7)

Abbreviations: CI, confidence interval; n, number; OR, odds ratio.

^a^ More than one site per patient possible.

^b^ Adjusted for sex, age, history of atopic dermatitis and contact allergy to fragrances (contact allergy to any of fragrance mix I, fragrance mix II, balsam of Peru, colophonium, lichen acids mix, hydroperoxide of limonene or hydroperoxide of linalool).

^c^ Scalp, ears or neck.

### 3.6 Concomitant contact allergies

Positive patch test reactions to allergens other than carvone were present in 24 of the 28 patients with carvone allergy. The prevalence of contact allergy to metals and preservatives in the Swedish baseline series did not differ between carvone‐positive and carvone‐negative patients (Table 2). However, contact allergy to gold was the most prevalent positive co‐reaction in the carvone‐positive group (39%, 11 of 28), whereas 13% (452 of 3452) reacted to gold in the carvone‐negative group (Table 3). The correlation between carvone and gold allergy was statistically significant (corrected OR (95% CI) 3.7 (1.7–8.1)).

**TABLE 2:** Concomitant contact allergies in patients with carvone contact allergy

	Carvone‐positive		Carvone‐negative			
Concomittant allergy to:	Total, *n*	With allergy to *n* (%)	Total, *n*	With allergy to *n* (%)	OR (95% CI)	
					Univariate	Multivariate^a^
Fragrances^b^	27	10 (37)	3274	667 (21)	2.1 (1.0–4.5)	1.8 (0.8–3.9)
Preservatives^c^	27	1 (4)	3242	300 (9)	0.4 (0.0–2.6)	0.3 (0.0–2.4)
Metals^d^	27	7 (26)	3207	644 (20)	1.3 (0.6–3.1)	1.0 (0.4–2.5)

Abbreviations: CI, confidence interval; n, number; OR, odds ratio.

^a^ Adjusted for sex age, and history of atopic dermatitis.

^b^ Fragrance‐related allergens: fragrance mix I, fragrance mix II, balsam of Peru, colophonium, lichen acids mix, hydroperoxide of limonene and hydroperoxide of linalool.

^c^ Paraben mix, methylchloroisothiazolinone/methylisothiazolinone, formaldehyde, diazolidinyl urea, quaternium‐15 and methyldibromoglutaronitrile.

^d^ Nickel(II)sulfate hexahydrate, cobalt(II)chloride hexahydrate and potassium dichromate.

**TABLE 3:** Contact allergies to single allergens

	Carvone‐positive		Carvone‐negative			
Substance, concentration %	Positive reactions, *n* (%)	All tested	Positive reactions, *n* (%)	All tested	OR (95% CI)	
					Univariate	Multivariate^a^
Fragrances/flavours						
FM I, 8.0	0 (0)	28	224 (6.4)	3497	–	–
FM II, 14.0	0 (0)	28	84 (2.4)	3497	–	–
Balsam of Peru, 25.0	4 (14.3)	28	208 (5.9)	3497	2.6 (0.9–7.7)	2.0 (0.7–5.9)
Colophony, 20.0	1 (3.6)	28	95 (2.79)	3497	1.3 (0.2–9.9)	0.9 (0.1–7.0)
Lichen acids mix, 0.03	0 (0)	28	25 (0.7)	3495	–	–
Hydroperoxides of limonene, 0.3	5 (17.9)	28	249 (7.1)	3497	2.8 (1.1–7.5)	2.6 (1.0–7.1)
Hydroperoxides of linalool, 0.1	2 (7.1)	28	275 (7.9)	3497	0.9 (0.2–3.8)	0.9 (0.2–3.8)
Metals						
Gold sodium thiosulfate, 2.0	11 (39.3)	28	452 (13.1)	3452	4.3 (2.0–9.2)	3.7 (1.7–8.1)
Nickel sulfate, 5.0	5 (17.9)	28	541 (15.7)	3452	1.2 (0.4–3.1)	0.9 (0.3–2.3)
Potassium dichromate, 0.5	1 (3.6)	28	138 (4.0)	3452	0.9 (0.1–6.6)	0.9 (0.1–6.8)
Cobalt chloride, 1.0^b^	1 (3.6)	28	139 (4.0)	3452	0.9 (0.1–6.5)	0.6 (0.08–4.7)
Preservatives						
MCI/MI, 0.215^b^	0 (0)	28	173 (5.0)	3488	–	–
Methyldibromo glutaronitrile, 0.5	1 (3.6)	28	94 (2.7)	3488	1.3 (0.2–9.9)	1.1 (0.1–8.5)
Paraben mix,^c^ 16.0	0 (0)	28	13 (0.4)	3488	–	–
Formaldeyde, 2.0	0 (0)	28	124 (3.6)	3488	–	–
Diazolidinylurea, 2.0	0 (0)	28	16 (0.5)	3496	–	–
Quaternium‐15, 1.0	0 (0)	28	31 (0.9)	3497	–	–

Abbreviations: CI, confidence interval; FMII, fragrance mix I; FMII, fragrance mix II; n, number; MCI/MI, methychloroisothiazolinone/methylisothiazolinone; OR, odds ratio.

^a^ Adjusted for sex, age, and history of atopic dermatitis.

^b^ From January 2021, cobalt chloride increased from 0.5% to 1% in pet and methychloroisothiazolinone/methylisothiazolinone (MCI/MI) from 0.02% to 0.215% in pet.

^c^ Methylparaben, ethylparaben, propylparaben and butylparaben.

Ten out of 28 carvone‐positive patients had one or more fragrance‐related co‐reaction. Table 3 shows that the most common was hydroperoxides of limonene (5 of 28, *p* = 0.047), although only a borderline association were found after multivariate analysis (OR (95% CI) 2.6 (1.0–7.1)). The majority (18 of 28) did not have a concomitant fragrance‐related allergy. Of these, 44% (8 of 18) had a clinically relevant allergy to carvone.

## 4 DISCUSSION

### 4.1 Prevalence

The five‐year prevalence of carvone was 0.79% in this study. This is in line with the prevalence of consecutive dermatitis patients of 0.78% reported in a recent large North American study,^13^ but lower than the prevalence of 2.8% found in an earlier European study.^14^ In another study from the same geographic area as the present study the carvone‐positive prevalence was 3.5%. However, the study mainly included dental patients and when examining consecutive dermatitis patients only the prevalence was 1.0%, which is more consistent with our result.^15^

In the literature, it has been discussed if the test concentration (5%) is marginally irritant.^14^ In the present study, doubtful test reactions were relatively common, while irritant reactions were rare. In our experience, with the present patch test preparation of carvone, strong reactions are rare, and not only the possibility of an irritation effect must be considered, but also the possibility of a suboptimal patch test concentration.

The annual prevalence increased slightly over the 5 years studied which could reflect a different or an increased exposure. The global mint essential oils market is expected to grow at a compound annual growth rate of 9.2% from 2019 to 2025 and to reach USD 330.0 million by 2025.^16^ New sources of exposure include flavoured snuff/snus, for example, spearmint taste.^17^

### 4.2 Relevance

In this study, detected carvone allergies were often found clinically relevant. Furthermore, in more than one‐fourth of the carvone‐positive patients, there was no suspicion of fragrance/flavour allergy before the detection of the clinically relevant allergy.

### 4.3 Affected anatomical site(s)

The overrepresentation of oral/lip and anogenital engagement, respectively, and underrepresentation of hand/arm engagement in the carvone allergic patients is in line with the recent North American study,^13^ but differs from the earlier European study in dermatitis patients were the majority^14^ had hand/arm engagement and none had anogenital engagement.^14^ Carvone is primarily related to products used orally unlike the fragrance mixes in the Swedish baseline series, and the likely sensitization route to carvone is through oral exposure.^15^ Thus, the large proportion of patients with intraoral/lip involvement was not unexpected considering carvone is a common flavour component in oral health products, foods, beverages (like tea), confectionery and tobacco products.

The association with anogenital disease is intriguing. As far as we know, it is not obvious with carvone exposure in this area; however, we cannot rule out that the allergen after oral intake becomes metabolized and is found in urine or faeces. Systemic exposure as a cause of anogenital symptoms is a less plausible explanation, but cannot be excluded.^18^, ^19^ Another hypothetical explanation may be lichen planus acting as a confounding factor. An association between oral lichen and carvone contact allergy has been reported.^15^, ^20^ A patient with genital changes may also have a current or a previous oral involvement.^21^

### 4.4 Concomitant contact allergies

Given the kinship between carvone and fragrances, one might assume that routine fragrance allergy markers also can act as a marker for carvone allergy. As carvone and limonene are chemically related, an association between carvone and oxidised limonene would not be surprising. However, in previous studies, only a small number of patients sensitised to carvone also reacted to oxidised limonene,^13^, ^15^ and in the present study only a weak association between hydroperoxides of limonene and carvone was found. Furthermore, a significant part of the carvone‐positive patients (64%) did not have a concomitant allergy to a fragrance/flavour substance, and 44% of those with a clinically relevant carvone allergy would have been missed if not tested with carvone. This indicates that test with the fragrance mixes, balsam of Peru or hydroperoxide of limonene are no good markers for carvone allergy and supports further studies to possibly include the allergen in the Swedish baseline series.

In an earlier study, concomitant allergy to sesquiterpene lactone mix was common,^8^, ^14^ but in our study and in the recent North American study, this was uncommon.^13^

An association between gold and carvone allergy was seen and the common factor could be oral exposure. Previous studies have shown that contact allergy to gold is associated with dental gold exposure and oral lichen.^22^, ^23^, ^24^, ^25^ The latter is analogous to a recent report of correlation between carvone allergy and oral lichen and suggests that stomatitis is associated with carvone allergy.^15^

## 5 CONCLUSION

Carvone is a common flavour component in several different products that we are exposed to on a daily basis which may reflect the significant fraction of relevant allergies found in this study. Our results imply that the fragrance/flavour‐related allergens in the baseline series are not good markers for carvone contact allergy. The results indicate that an inclusion of carvone in the baseline series even if the contact allergy prevalence is below 1% should be considered.^25^ Further study of the optimal test concentrations is warranted.

### 5.1 Limitations

Retrospective design, possible incorrect recorded data and interpretation of clinical relevance, and albeit a large number of patients a limited number of carvone‐positive patients.

## AUTHOR CONTRIBUTIONS

**Johanna Enberg:** Writing – original draft; investigation; data curation; software. **Nils Hamnerius:** Conceptualization; software; data curation; methodology; formal analysis. **Liv Kroona:** Validation; supervision. **Cecilia Svedman:** Conceptualization; validation; project administration; resources; supervision.

## CONFLICT OF INTEREST

The authors have no pertinent conflict of interests to declare.

## Data Availability

The data that support the findings of this study are available on request from the corresponding author. The data are not publicly available due to privacy or ethical restrictions.
